# Ukumela impilo randomised trial: preliminary findings of height-adjustable sit-to-stand workstations on health outcomes of South African office workers

**DOI:** 10.1186/s13104-023-06642-2

**Published:** 2023-12-07

**Authors:** Merling Phaswana, Philippe Jean-Luc Gradidge

**Affiliations:** Department of Exercise Science and Sports Medicine, Faculty of Health Sciences, University of the Witwatersrand, Johannesburg, South Africa

**Keywords:** Sedentary behaviour, Cardiometabolic outcomes, Height adjustable sit-to-stand workstation, Office-based workers, South Africa

## Abstract

**Background:**

The prevalence of sedentary behaviour has concurrently risen with multiple cardiometabolic risk markers independent of physical activity levels. Office-based workers accumulate the highest levels of sitting time during occupational times. This study aims to investigate the short-term effects of using height-adjustable sit-to-stand workstations on cardiometabolic risk markers of office-based workers in South Africa.

**Results:**

Sixty-two office-based workers were randomized into intervention (n = 44), and the control group (n = 18). Small improvements were observed in BMI, blood pressure, and cholesterol levels in this cohort.

**Conclusion:**

This preliminary investigation confirms that short-term height-adjustable sit-stand interventions are effective in reducing workplace sitting time and selected health outcomes. South Africa has been attributed with the highest burden of obesity in Sub-Saharan Africa, as a result, there is a need to implement long-term workplace intervention to reverse these implications.

**Trial registration:**

Pan African Clinical Trial Registry, PACTR201911656014962 on the 12th of November 2019.

## Introduction

Sedentary behaviour is defined as prolonged sitting, lying down, or low-energy activities of less than 1.5 metabolic equivalents [1], and is an important public health issue [2]. Sitting for more than 6 h per day, defined as prolonged sitting by WHO, increases the risk of premature death, even for individuals who maintain a regular exercise routine [3]. The modern-day workforce is predominantly office-based and spends ≥ 8 h in uninterrupted sitting despite its concerning implications [4]. A recent investigation has demonstrated that the South African workforce experiences prolonged sitting to a similar extent as observed in other settings [5]. This is particularly concerning given that low and middle-income countries (LMICs) like South Africa have the highest rates of obesity and non-communicable diseases (NCDs) [6]. In a systematic review that investigated the effects of sedentary behaviour reduction on cardiometabolic risk markers among office-based workers, nine were deemed extremely promising, while ten were categorized as non-promising [7]. This systematic review suggests that interrupting prolonged sitting time with a small amount of energy expenditure may improve overall health in office-based workers.

A recent three-arm randomized controlled trial investigating the effectiveness of multifaceted strategies of behavioural change with and without a height-adjustable desk, found notable mean change improvements in daily sitting time (-21.2 ± 116.7; -11.4 ± 106.9) mg, body mass index (-0.02 ± 1.1); 0.1 ± 1.6) kg.m^2^, and systolic blood pressure (-2.1 ± 11.3; -2.1 ± 11.8) mmHg in both short (≤ 12 months) and long term (≥ 12 months) follow up [8]. Therefore, to mitigate the negative health effects associated with prolonged sitting in the workplace, it is recommended to introduce strategies to interrupt sitting time [4]. Similarly, multicomponent interventions to reduce sedentary behaviour and cardiometabolic health in the workplace have shown to be effective particularly those involving height-adjustable workstations [9, 10]. Although existing evidence suggests that environmental strategies such as height-adjustable sit-to-stand interventions have the potential to reduce occupational sitting time and improve overall health in high-income countries (HICs) [4, 11], it is difficult to generalise these findings to low and middle-income countries (LMICs). Furthermore, there is currently no evidence of the implementation of environmental strategies such as height-adjustable sit-to-stand interventions in South African and African workplaces, including university settings [5]. The purpose of this randomized controlled study was to evaluate the effectiveness of a 12-week height-adjustable sit-to-stand intervention on sedentary behaviour and cardiometabolic health outcomes among office-based workers in South Africa.

## Methods

### Study design and participants

This randomised controlled trial was conducted at the University of the Witwatersrand, Johannesburg, and a credit bureau, Johannesburg, South Africa. All participants provided written consent and the criteria for inclusion in the study had been previously reported [1]. Ethical clearance was granted by the Human Research Ethics Medical Committee from the University of the Witwatersrand (ethics certificate number M190224).

### Intervention

A single-blinded randomized controlled trial (RCT) was conducted with a total of 122 participants that were randomly assigned to either the intervention or control group. The group allocation was conducted by a qualified biostatistician independent from the core research team to randomly assign participants into control and intervention. The intervention group consisted of (n = 62, 51%) participants, while the control group had (n = 60, 49%) participants. Participants in the intervention group were provided with a height adjustable sit-to-stand workstation (JUMBO DeskStand™, DeskStand, South Africa) as previously described in the protocol [12] and pilot study [5]. The researchers modified participants’ existing workstations by installing a height-adjustable sit-to-stand workstation on top of their desks, which was individually configured for proper ergonomics. Participants were provided with information sheets and trained on how to effectively use the workstation when in the sitting and standing positions. Based on existing evidence, we initially recommended, short intermittent bouts of standing activity lasting at least 10 min and were encouraged, to progress to longer bouts of at least 30 min every hour for the duration of the intervention [13]. Participants were encouraged to interrupt their sitting time by accumulating bouts of standing activity with an emphasis on reducing sitting time. During the study, the researchers regularly visited the participants to assess the effectiveness of the height-adjustable sit-to-stand workstation and encourage them to interrupt prolonged sitting. Additionally, the participants received regular communication regarding the benefits of interrupting their sitting time.

The control group participants continued to use their traditional desks and were informed verbally about the negative health effects of prolonged sitting. The researcher did not interact with the control group participants during the intervention period, except to collect baseline and follow-up data at 12 weeks.

### Measurements

Measurements were taken at baseline and the 12-week follow-up for all participants. Participants self-reported their age, gender, level of education, and smoking status. Body weight was measured using a digital scale (Omron HN288, Japan) [14], height was measured using a stadiometer (Seca 123, USA), and obesity was defined as a body mass index (BMI) of ≥ 30 kg.m^2^ [14]. Waist circumference was measured using a measuring tape (Gulick, USA), and central obesity was defined as a waist circumference greater than 94 cm for males and 80 cm for females [15]. Blood pressure was measured using a monitor (Omron M7 Intelli IT (HEM-7322T-E), Omron, Kyoto, Japan), and hypertension was defined as systolic blood pressure ≥ 140 mm Hg and diastolic blood pressure ≥ 90 mmHg, or a history of hypertension or use of hypertension medication [16]. Blood samples were taken to measure random glucose, glycated haemoglobin (HbA1c), total cholesterol, high-density lipoprotein (HDL) cholesterol, low-density lipoprotein (LDL) cholesterol, and triglycerides as previously described in the protocol [1]. A diagnosis of diabetes was defined as random glucose ≥ 11.1 mmol/L, HbA1c ≥ 6.5%, use of antidiabetic medication(s), or history of diabetes. The AX3 accelerometer (Newcastle-upon-Tyne, United Kingdom) was used to evaluate sleep, sedentary behaviour, light physical activity (LPA), and moderate-to-vigorous physical activity (MVPA) [17].

### Statistical analysis

Statistica version 13 (StataSoft Inc., Tulsa, OK, USA) was used for analysis. The normality of the data was determined using the Shapiro-Wilk test and histograms. Data that was normally distributed was presented as mean ± standard deviation, or frequency (percentage), while skewed data was presented as median (interquartile range). The differences between baseline and 12-week are presented as effect sizes using Cohen’s d. The differences between study groups were determined using dependent t-tests. Independent t-tests and analysis of covariance (ANCOVA) were used to determine the differences in absolute changes in outcomes of interest between the control group and intervention groups. The dependent t-test was performed to determine mean changes between the intervention and controls. The effect sizes were interpreted as large (≥ 0.8), moderate (0.4 to 0.8), small (0.2 to 0.4), and trivial (< 0.2). Significance was set at *p* < 0.05.

## Results

**Fig. 1:**
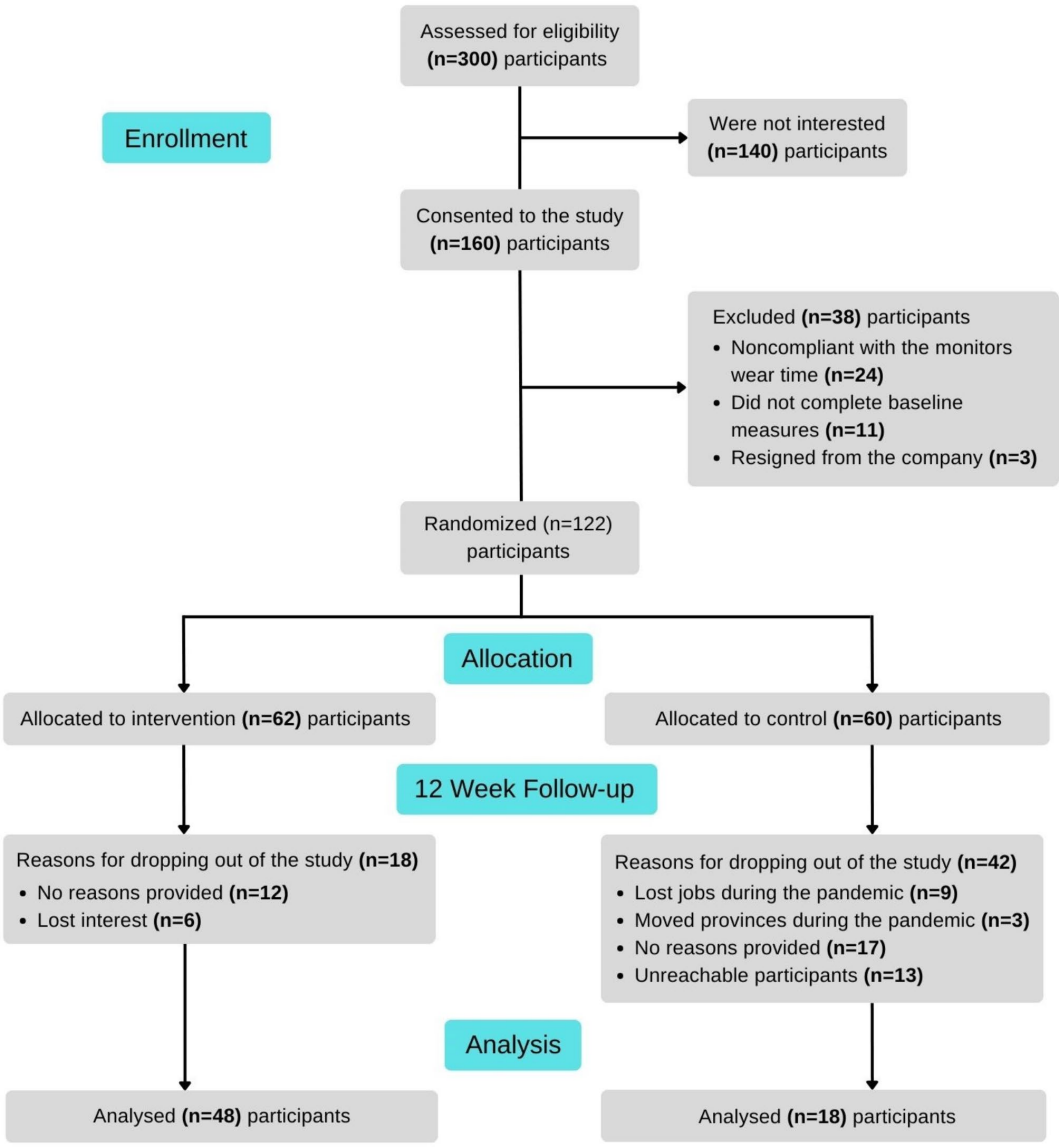
Flow diagram of participants through randomised controlled trial

Figure 1 shows the flow of participants involved in the study. One hundred and sixty participants provided written consent to participate in this study, however, 38 participants were excluded from the study due to non-compliance, incomplete measures, and withdrawal from the study. One hundred twenty-two participants were randomised into the intervention group (n = 62) and control group (n = 60). We observed a significant drop out of the study for the following reasons, provided no reasons (n = 29), loss of interest (n = 6), unreachable (n = 13), retrenched (n = 9) and relocated or moved provinces (n = 3, %) The final sample of 62 were randomized into the intervention n = 4 (71%) and control n = 18 (29%) groups, respectively.

### Subject characteristics at baseline

Table 1 presents the mean age of the participants in the study was 40.32 ± 10.12 years, and were mostly female (n = 49, 79%). High mean values for BMI were observed in both the intervention group (31.32 ± 7.03 kg.m^2^) and the control group (28.13 ± 4.21; kg.m^2^), *p* < 0.001 at baseline. The percentages for overweight and obesity were (n = 21, 33.87%) and (n = 29, 46.77%), respectively. The systolic and diastolic BP of the intervention (112.55 ± 14.05 and 119.81 ± 14.88; <0.001 mmHg) and the control group were (81.78 ± 8.75 and 81.56 ± 9.42 mmHg), *p* < 0.001. Hypertension was (n = 38, 61.29%) and type 2 diabetes was determined to be (n = 25, 40.32%) in this cohort office-based workers. Sedentary behaviour was 97.52 (80.51- 109.74 min/day) in the intervention group and 79.65 (53.58–90.67 min/day) in the control group, *p* < 0.001 at baseline. Median MVPA values were 24.64 (11.68–39.01 min/day) in the intervention group and 25.30 (9.10-51.08 min/day) in the control group, *p* < 0.00001.

**Table 1:** Demographic and baseline characteristics of study participants

	Combined sample (n = 62)	Intervention group (n = 44)	Control group (n = 18)	***p***-value for model
Age (years)	40.32 ± 10.12	41.88 ± 9.37	36.31 ± 11.15	0.247
Female (%)	49 (79.03)	38 (77.55)	11 (22.45)	0.027
Current smokers (%)	12 (100)	7 (58.33)	5 (41.67)	0.134
**Education Level**				
Completed high school (%)	11 (18.33)	67 (63.64)	4 (36.36)	< 0.001
College diploma (%)	15 (25.00)	8 (53.33)	7 (46.67)	< 0.001
University degree (%)	14 (23.33)	10 (71.43)	4 (28.57)	< 0.001
Postgraduate qualification (%)	20 (33.33)	17 (85.00)	3 (15.00)	< 0.001
**Cardiometabolic Outcomes**				
BMI (kg/m^2^)	30.39 ± 6.47	31.32 ± 7.03	28.13 ± 4.21	< 0.001
WC (cm)	88.56 ± 12.23	89.93 ± 11.87	85.22 ± 12.80	< 0.001
Overweight (%)	21 (33.87)	15 (24.19)	6 (9.68)	< 0.001
Obesity (%)	29 (46.77)	21 (33.87)	8 (12.90)	< 0.001
SBP (mmHg)	114.65 ± 14.56	112.55 ± 14.05	119.81 ± 14.88	< 0.001
DBP (mmHg)	81.72 ± 8.87	81.78 ± 8.75	81.56 ± 9.42	< 0.001
Hypertension (%)	38 (61.29)	27 (43.44)	11 (17.74)	< 0.001
RBG (mmol/L^− 1^)	6.70 ± 2.71	6.81 ± 3.11	6.43 ± 1.36	< 0.001
HbA1c (%)	6.11 ± 1.15	6.16 ± 1.29	5.98 ± 0.67	< 0.001
Diabetes (%)	25 (40.32)	16 (25.81)	9 (14.51)	< 0.001
Triglycerides (mmol/L^− 1^)	1.52 ± 0.66	1.53 ± 0.66	1.49 ± 0.66	< 0.001
HDL (mmol/L^− 1^)	1.33 ± 0.34	1.37 ± 0.36	1.22 ± 0.26	0.082
LDL (mmol/L^− 1^)	1.70 ± 1.25	1.63 ± 1.33	1.88 ± 1.01	< 0.001
Total Cholesterol (mmol/L^− 1^)	4.09 ± 1.23	4.25 ± 1.20	3.69 ± 1.25	< 0.001
**Accelerometry data (median IQR)**				
Sleeping time (mins/day)	352.19 (233.51–392.23)	364.89 (243.24–393.51)	319.05 (189.67–389.95)	< 0.001
Sedentary time (mins/day)	89.12 (73.24–107.51)	97.52 ( 80.51- 109.74)	79.65 (53.58–90.67)	< 0.001
LPA (mins/day)	111.49 (84.96–130.08)	111.49 (96.22–133.76)	103.72 (67.72–129.24 )	< 0.001
MVPA (mins/day)	24.64 (10.27–39.23)	24.64 (11.68–39.01)	24.18 (9.15–65.43)	< 0.001

Body Mass Index (BMI); Diastolic blood pressure (DBP); Systolic blood pressure (SBP); Waist Circumference (WC); Random blood glucose (RBG); Glycated haemoglobin (HbA1c); high-density lipoprotein cholesterol (HDL); low-density lipoprotein cholesterol (LDL); Light physical activity (LPA); moderate to vigorous physical activity (MVPA)

### Effectiveness of a height adjustable sit-to-stand intervention

Table 2 presents changes between baseline and follow-up for cardiometabolic health outcomes with free-living sedentary behaviour and physical activity data. Sedentary behaviour was reduced in the intervention group (-9.3 ± 37.13 min/day) while showing an increase in the control group (7.66 ± 36.44 min/day). Light physical increased in the intervention group (4.14 ± 51.04 min/day) and decreased in the control group (-14.71 ± 52.03 min/day) from baseline to follow-up. Moderate to vigorous physical activity increased in both the intervention (3.35 ± 20.86 min/day) and the control groups (6.36 ± 25.32 min/day) respectively. When considering BMI and total cholesterol measures, we observed trivial effects of (d= -0.11 kg.m^2^) and (d=-0.11 mmol/L-1). Similar trivial effects were observed in most cardiometabolic outcomes. Small effects of were only observed with diastolic blood pressure (d = 0.26 mmHg) and light physical activity (d = 0.26 min/day) in the intervention.

**Table 2:** Changes in cardiometabolic health outcomes with free-living sedentary behaviour and physical activity data

	Intervention (n = 44)	Control (n = 18)	Intervention vs. Control		
	Mean change (standard deviation)	Mean change (standard deviation)	Mean change (standard deviation)	Effect size (*d*)	***P***-value
BMI (kg/m^2^)	-0.52 ± 2.72	0.55 ± 2.98	1.08 ± 2.80	-0.11	0.005
WC (cm)	0.07 ± 7.42	-0.06 ± 7.41	-1.32 ± 7.36	0.11	0.162
SBP (mmHg)	2.70 ± 13.33	-4.64 ± 10.55	-1.86 ± 12.83	0.06	0.258
DBP (mmHg)	-0.24 ± 9.51	0.86 ± 9.56	-1.37 ± 9.50	-0.26	0.260
RBG (mmol/L-1)	-0.40 ± 1.62	-0.72 ± 1.45	-0.80 ± 1.60	0.13	< 0.001
HbA1c (%)	0.25 ± 1.46	0.10 ± 1.03	-1.58 ± 1.51	0.08	< 0.001
TC (mmol/L^− 1^)	-0.02 ± 1.02	0.15 ± 1.76	-1.32 ± 1.37	-0.11	< 0.001
Triglycerides (mmol/L^− 1^)	0.10 ± 0.83	0.21 ± 1.33	-1.43 ± 1.11	0.01	< 0.001
HDL (mmol/L^− 1^)	0.02 ± 0.32	0.04 ± 0.36	-1.32 ± 0.57	0.08	< 0.001
LDL (mmol/L^− 1^)	0.36 ± 1.20	0.02 ± 1.45	-1.42 ± 1.59	0.11	< 0.001
Sleeping time (mins/day)	-8.89 ± 136.32	-51.39 ± 94.31	-22.52 ± 126.39	0.11	0.166
Sedentary time (mins/day)	-9.3 ± 37.13	7.66 ± 36.44	-3.08 ± 37.54	0.06	0.520
LPA (mins/day)	4.14 ± 51.04	-14.71 ± 52.3	2.62 ± 51.78	-0.26	0.691
MVPA (mins/day)	3.35 ± 20.86	6.36 ± 25.3	5.52 ± 22.10	0.13	0.054

Body Mass Index (BMI); Diastolic blood pressure (DBP); Systolic blood pressure (SBP); Waist Circumference (WC); Random blood glucose (RBG); Glycated haemoglobin (HbA1c); high-density lipoprotein cholesterol (HDL); low-density lipoprotein cholesterol (LDL); Light physical activity (LPA); Moderate or vigorous physical activity (MVPA)

## Discussion

This study evaluated preliminary findings of a longitudinal randomized controlled trial (RCT) to address sedentary behaviour and cardiometabolic risk markers in a cohort of South African office-based workers. It is worth noting that this intervention focussed solely on improving cardiometabolic health by using height-adjustable desk to reduce sitting time during work hours. This current study demonstrates that a height-adjustable sit-to-stand workstations are effective in reducing sedentary behaviour and improving cardiometabolic outcomes in a cohort of South African office workers over a 3-month, follow-up period. These data are important for informing further longitudinal studies of this environmental modification in the workplace.

Our findings show that sedentary behaviour decreased in the intervention (-9.3 min/day) and increased in the control group (7.66 min/day) when measured with accelerometry devices respectively. These results are consistent with those of a previous intervention study that demonstrated significant reductions in sedentary behaviour in the workplace in HICs [10]. The findings of the current study are similar to those of a recent RCT [8], which found that sedentary behaviour deceased in both the behavioural change with (-13.00 (-29.5 to 3.6 min) and without a height adjustable desk (-74.3 (-90.8 to -57.7 min) interventions when compared to the control group in 3 months follow up. An improvement in light physical activity and MVPA was observed in the group using the height-adjustable desk. Although bouts of standing were not quantified in the present study in comparison to previous studies that have demonstrated that interrupting sitting time by standing increases overall physical activity [9, 10]. Therefore, substituting prolonged sitting time with comparable amounts of light or moderate activity may improve health [18].

An encouraging finding of this study is that small and trivial improvements were observed in most cardiometabolic risk markers is in agreement with a recent systematic review and meta-analysis [7]. For instance, in the current study, we observed small effects on health outcomes such as BMI (d=-0.11) kg.m^2^, blood pressure (d=-0.26) mmHg, and cholesterol levels (d = 0.11) mmol/L^− 1^ in 3 months. It is important to note that the changes observed in this study were relatively small and may not be statistically significant due to a relatively small sample size [7, 11]. Despite the paucity of data in LMICs, our study supports the use of height-adjustable sit-to-stand interventions in reducing sedentary behaviour and improving cardiometabolic outcomes among South African office-based workers [5]. However, modifying the existing workplace environment by introducing a height-adjustable sit-stand workstation not be enough to significantly reduce sedentary behaviour and improve health outcomes.

Systematic reviews investigated the effectiveness of sedentary behaviour reduction workplace interventions on cardiometabolic risk markers suggesting that both short-term and long-term interventions are effective in reducing prolonged sitting [4, 7, 11]. It is not clear which cardiometabolic risk markers improve with sedentary behaviour interventions. Interestingly previous multicomponent long term interventions reduced daily sitting time significantly. Healy et al. [9] reported a 44 min drop in 231 office workers, Edwardson et al. [19] found similar reductions of 41 min in a sample of 143 office workers, Pereira et al. [20] found a decrease of 60 min in a sample of 630 office workers and Edwardson et al. [8] found a decrease of 22–62 min in a sample of 547 office workers. This suggests that a combination of environmental strategies such as height-adjustable sit-to-stand workstations and additional strategies such as education, motivation and coaching might be more effective in reducing sedentary behaviour and improving overall health. Further research is needed to identify and implement effective long-term sedentary behaviour strategies aimed at achieving sustained behaviour change in the workplace, particularly in LMICs such as South Africa. The strengths of this study include the robust nature of the methods used and the positive findings that can be used to inform further studies on office workers.

## Conclusion

This investigation confirms that short-term height-adjustable sit-stand interventions are effective in reducing workplace sitting time and selected health outcomes. While the effect sizes were small, the results are encouraging, and they suggest that even short-term interventions can have a positive effect on health. Further research is warranted to validate these findings and to explore the long-term impact of a sit-to-stand workstation on reducing sedentary behaviour and enhancing the health outcomes of office workers in South Africa.

### Limitations

There are important implications to these preliminary findings that should be recognized. More than 60% of the participants dropped out of the current study which reduced the size of the study sample and may limit the generalizability of the findings. The authors hypothesized that this high drop-out could be attributed to the COVID-19 pandemic implications experienced during the 12-week trial as limited movement and companies moving to full remote (working from home) at the time of the study. Another limitation of the study was that it was conducted during the Covid-19 pandemic when movement restrictions a shift to full remote work and the closing of companies were imposed on South African workplaces, which may have influenced the large dropout rate.

## Data Availability

The datasets used in this study are available from the from the corresponding author (MP) on request.
