# Correction: Ukumela Impilo randomised trial: preliminary findings of height-adjustable sit-to-stand workstations on health outcomes of South African office workers

**DOI:** 10.1186/s13104-024-06733-8

**Published:** 2024-03-14

**Authors:** Merling Phaswana, Philippe Jean-Luc Gradidge

**Affiliations:** Department of Exercise Science and Sports Medicine, Faculty of Health Sciences, University of the Witwatersrand, Johannesburg, South Africa

**Correction: BMC Research Notes (2023) 16:361**

10.1186/s13104-023-06642-2

Following publication of the original article, we were informed that author Philippe Jean-Luc Gradidge’s given and family names were incorrectly captured in the article XML, resulting to an incorrect citation.

The author’s given names are: Philippe and Jean-Luc.

The author’s family name is: Gradidge.

The original article has now been revised.

